# Rapid hot-injection as a tool for control of magnetic nanoparticle size and morphology

**DOI:** 10.1039/d1ra02977k

**Published:** 2021-06-09

**Authors:** Magdalena Kulpa-Greszta, Anna Tomaszewska, Andrzej Dziedzic, Robert Pązik

**Affiliations:** Faculty of Chemistry, Rzeszow University of Technology Aleja Powstańców Warszawy 12 35-959 Rzeszow Poland mkulpa@ur.edu.pl; Department of Biotechnology, Institute of Biology and Biotechnology, College of Natural Sciences, University of Rzeszow Pigonia 1 35-310 Rzeszow Poland; Department of Spectroscopy and Materials, Institute of Physics, College of Natural Sciences, University of Rzeszow Pigonia 1 35-310 Rzeszow Poland

## Abstract

The rapid hot-injection (HI) technique was employed to synthesize magnetic nanoparticles with well-defined morphology (octahedrons, cubes, and star-like). It was shown that the proposed synthetic approach could be an alternative for the heat-up and flow hot-injection routes. Instant injection of the precursor to the hot reaction mixture (solvent(s) and additives) at high temperatures promotes fast nucleation and particle directional growth towards specific morphologies. We state that the use of saturated hydrocarbon namely hexadecane (sHD) as a new co-solvent affects the activity coefficient of monomers, forces shape-controllable growth, and allows downsizing of particles. We have shown that the rapid hot-injection route can be extended for other ferrites as well (ZnFe_2_O_4_, CoFe_2_O_4_, NiFe_2_O_4,_ and MnFe_2_O_4_) which has not been done previously through the HI process before.

## Abstract 2

Rapid hot-injection can be used for precise control of magnetic particle shape.

## Graphical abstract

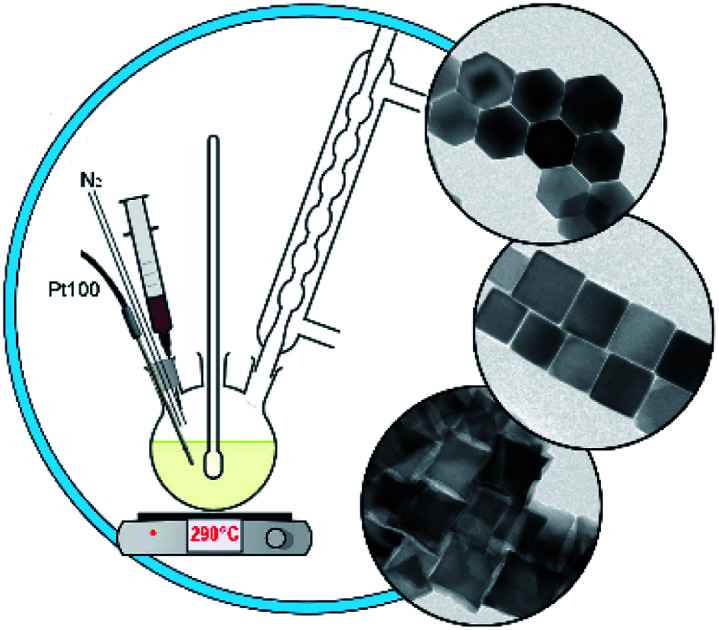

## 1. Introduction

The huge interest of many research groups in the development of magnetic iron oxide nanoparticles (MNPs) is a direct consequence of their exceptional, strongly size and morphology dependent magnetic properties as well as acceptable biocompatibility and sufficient biodistribution.^1–3^ Hence MNPs can be exploited in a variety of biomedical applications both at diagnostic and therapy levels such as in magnetic resonance imaging (MRI),^4,5^ localized drug delivery,^6–8^ tissue regeneration^9^ or hyperthermia.^10,11^ Several interesting studies devoted to the synthetic issues of MNPs were already published. The authors emphasize that it is of absolute necessity to control the MNP shape (uniformity and preferably cubic particles), size (optimal range for magnetism and biological applications), distribution (predictable properties), particle state in a colloidal suspension (agglomeration/aggregation as a limiting factor for the magnetic interparticle interactions, biodistribution), and chemical composition (magnetic properties and biocompatibility). Moreover, a conscious decision regarding the choice of protecting/stabilizing/targeting/conjugating organic molecules has to be taken.^1,12–14^ Therefore, it is still a real challenge to develop MNPs precisely tailored for biological systems since multiple parameters can critically affect both the physical and biological properties of MNPs. One can easily find a plethora of scientific articles dedicated to synthetic approaches toward MNPs starting from simple and easy techniques like co-precipitation,^15^ sol–gel,^16^ microemulsion,^17^ hydrothermal.^18^ Special attention should be paid to the thermal decomposition method carried out in the high boiling point organic media.^19^ Mainly, route based on thermal decomposition of metalorganic precursors in the organic phase can be subdivided into two main groups: so-called heat-up^20–26^ and hot-injection^27–29^ synthesis. The heat-up technique involves continuous heating of the reaction mixture (one-pot, precursors, solvents and additives) until the desired temperature is reached. Afterward, decomposition of the precursor occurs leading to the nucleation and growth of particles (continuous processes).^19^ Whereas the hot-injection relies on the immediate injection of the precursor solution (cold) directly to the heated solvent with additives (hot). Rapid precursor decomposition results in the burst nucleation and further particle growth. The latter technique was developed for the preparation of quantum dots (QDs).^30^

A lot of effort has been made to improve the heat-up approach toward shape and size controlled MNPs by using as a precursor iron acetylacetonate and organic solvents. Due to the use of liquids with high boiling temperatures it was mandatory to find optimal reaction parameters and an appropriate solvent as the reaction medium. It was shown that the solvent type, *i.e.* benzyl ether,^23,31^ phenyl ether,^20^ dioctyl ether, 1-octadecene,^32^ eicosene, hexadecanol and docosene^33^ can greatly affect the particle morphology and size.^34^ However, another important issue was found in the thermal stability of organic liquids at high temperature or directly at the boiling point. The most commonly used are ethers especially benzyl ether (BE) which is prone to degradation into benzaldehyde (BA) and benzyl benzoate (BB). Unfortunately, even the stock solution of BE can contain both compounds. That leads to temperature instability resulting in a lack of control over process parameters and difficulties with product reproducibility.^9,35^ One of the possible ways to avoid that is to prepare a solvent mixture with the addition of highly non-polar co-solvents like octadecene, tetradecene. On the other hand, complete exchange of the BE is not possible since iron acetylacetonate has strictly limited solubility in non-polar liquids.^35^

Following the Wulff construction, first proposed by Gibbs and re-explored by Wulff, during the growth process nanoparticle will tend to assume an equilibrium final shape with minimized surface free energy.^36^ Thus, for isotropic materials polyhedral or spherical morphologies are generally favoured whereas for anisotropic compounds the Wulff approach has to be applied to determine the preferred particle shape. For the engineering of nanoparticles with defined properties, it is absolutely of high importance to consciously control the particle growth towards desired shapes. Additives/capping agents are a group of chemicals that have different binding ability to nanomaterials surface. The ligand can be specific or non-specific towards certain crystallographic facets/directions. Capping agent efficiently reduces surface energy and blocks the transport of monomers to the particle surface (restricted diffusion). If adsorption occurs at a specific facet, growth in this direction is inhibited and shape can be controlled. In the case of ferrites, three main morphologies are distinguished: cubic enclosed by {100}, octahedrons through {111}, and rhombic dodecahedrons by {110},^37^ respectively. As a matter of fact, to tailor MNPs morphology oleic acid (OA) and oleylamine (OAm) are broadly used.^1^ The role of an OA is extremely crucial since it takes part in the reaction of intermediate iron oleate complex formation and helps in partial reduction of the Fe^3+^ into Fe^2+^.^22^ Moreover, the OA adsorbs in a specific way on a {100} crystallographic facets favoring growth towards cubic particles. OA is also regarded as a protective ligand against aggregation through the formation of the hydrophobic layer around MNPs.^34,35^ Change in the additives chemical character and their ratio will affect nanoparticle shape greatly. Co-addition of the OAm, less polar in contrast to the OA, allows downsizing and facilitates the reduction of the iron in a greater manner than OA.^34^ However, one has to take into account that due to the non-specific binding of OAm control over a particle morphology might be difficult. It means that the concentration of both additives has to be always optimized.^34,38^ Usually, a typical heat-up procedure involves at least two main steps with different heating rates. The first one up to 200 °C to induce the formation of the iron oleate monomers (source of nuclei) from iron acetylacetonate precursor. The second step at/or near the boiling point of the solvent to force decomposition and tune the particle growth process.^35^ Through a change of the heating rate, it is possible to control the rapidity of monomer formation (exponential increase with temperature) and growth of nanoparticles to some extent.^1,25,39^

Surprisingly, in the context of the MNPs synthesis and morphology control *via* hot-injection route not much has been done and scarce information can be found in the specialised literature.^29,40–44^ The mechanism of the hot-injection was described in detail by Kwon and Hyeon.^44^ They reported that the role of the injection step is to deliver a very high level of supersaturation at the beginning of the reaction. After that formation of nanostructures occurs instantly leading to uniform particle growth. The way how the precursor is added to the reaction mixture and how nucleation occurs makes a huge difference between the heat-up method where nucleation and growth is a continuous process. However, the main drawback of the hot-injection emphasized by Wu *et al.* relies on mass-scaling and difficulties with control of temperature gradients due to the introduction of cold precursor to hot mixture.^19^

What is of great meaning for our paper is that Ho *et al.*^29^ have shown that by using flow hot-injection it is possible to control the shape of particles. In their approach, iron acetylacetonate precursor was added through the constant flow-injection (2.5–20 ml h^−1^). Moreover, the essence of the hot-injection technique in achieving a rapid supersaturation state has been somewhat lost – still rapid nucleation occurs but in a quasi-continuous way since even portions of a precursor are delivered over time. Thus, the mechanism of the particle formation, in this case, reminds greatly a seed-mediated process since the nuclei are formed continuously and growth occurs directly on the particle seeds.^21^ Standard chemicals were used *i.e.* BE as a solvent, OA, and OAm as additives. While 1,2-tetradecanediol was added to increase monomer activity through change the polar character of the reaction mixture. What is also important to note, is that the cubic shape of MNPs was obtained after more than 1 hour of synthesis duration. Thus, the mechanism of the particle shape formation must differ significantly. Taking into account that the BE can undergo detrimental changes over time at boiling point temperature it is highly likely that some reproducibility issues might occur upon protocol repetition.^35^ Nevertheless, the presented results were promising and provided additional possibility in an attempt to control the MNPs particle morphology by the slow, flow-injection route.

The ultimate goal of our work was to redefine the rapid hot-injection approach towards shape-defined MNPs *i.e.* Fe_3_O_4_. We state a hypothesis that the particle size and shape can be controlled by the addition of a strongly non-polar, monomer limiting activity saturated hydrocarbon (hexadecane sHD) as a co-solvent. The effect of the reaction temperature as well as the dependence of the concentration of precursor (iron acetylacetonate), additives (OA), and sHD was studied in detail. Our synthetic strategy has been further broadened for other ferrite examples (ZnFe_2_O_4_, CoFe_2_O_4_, NiFe_2_O_4_, MnFe_2_O_4_).

## 2. Experimental section

### 2.1 Synthesis of iron oxide nanoparticles

The synthetic part has been divided into three main strategies: (I) standard heat-up approach inspired by Kim *et al.*^23^ in which product was used as a reference sample and to test the effect of reaction temperature, (II) rapid hot-injection process and its optimization as well as (III) flow hot-injection technique to contradict some drawbacks and show further possibilities.

#### (I) Heat-up approach towards MNPs

Kim *et al.*^23^ procedure was adopted by us to prepare reference materials and to study the temperature effect on particle morphology. The main reason for choosing this protocol was its simplicity: one heating step with a fast temperature ramp (20 °C min^−1^) and high reproducibility of the products. As a precursor 2 mmol of iron(iii) acetylacetonate (Fe(acac)_3_; 99.7%, Thermo Fischer Scientific, Germany) were used, 1.5 ml of oleic acid (4.3 mmol, 90%, Sigma Aldrich, Poland) as a key and highly specific additive as well as 10 ml of dibenzyl ether (98%, Sigma Aldrich, Poland) as a solvent. All necessary manipulations with chemicals were performed in an acrylic glove box (GS Glove Box Systemtechnik GMBH P10R250T2) equipped with an automatic gas pressure control using a nitrogen gas inert atmosphere (N_2_ 99.999%, Linde, Poland) for protection. The iron precursor together with oleic acid were dissolved in BE using a three-neck glass flask equipped with a reflux column, gas line (a constant flow of N_2_), mechanical stirrer, and Pt-100 sensor connected to the temperature controller (LTR 2500, Juchheim, Germany). The reaction mixture was degassed for one hour at room temperature under a constant flow of inert gas. Afterward, the solution was heated to the desired temperature (270–290 °C) and kept for 30 minutes. The resulting black-dark brown product was purified by centrifugation–washing cycles (eight times) using ethanol solution (96%, Chempur, Poland) and redispersed as a stock MNPs suspension for further characterization.

#### (II) Rapid hot-injection procedure in the synthesis of MNPs

In this case, two alternatives were performed. First the rapid injection of the iron precursor into a hot mixture of BE containing OA (1.3–1.7 ml, 3.7–4.85 mmol) and the second where hexadecane (sHD, 99%, Sigma Aldrich, Poland) was used as an additional co-solvent with different proportions to the BE on already optimized OA content.

Typically, 7.6 ml of BE were mixed in a three-neck glass flask with 1.5 ml of OA and degassed under inert gas flow for 1 h at room temperature. After that solution was heated to 290 °C (below BE boiling point) under mechanical stirring and 2.4 ml of earlier prepared iron precursor (2 mmol of Fe(acac)_3_) in BE were rapidly injected through the syringe. The reaction mixture was kept for 30 minutes at 290 °C under gas flow. The obtained product was purified in the same manner as described in the previous part.

In the alternative protocol with sHD as a co-solvent 7.6 ml of BE, 1.5 ml of OA (4.3 mmol), and 3 ml of sHD (10 mmol) were mixed in a three-neck flask, degassed, and heated to 290 °C under stirring and inert gas flow. Again 2 mmol of Fe(acac)_3_ were dissolved in 2.4 ml of BE and rapidly injected into the hot reaction mixture. After 30 minutes at 290 °C, the reaction was stopped and the product was separated and purified as described above. Different quantities of sHD (5–12 mmol) and OA (1.5–1.7 ml) as well as an iron precursor (1.5–2.5 mmol) were used to track the effect of co-solvent, additive, and precursor concentration.

In the case of fabrication of the mixed ferrites *i.e.* ZnFe_2_O_4_, CoFe_2_O_4_, NiFe_2_O_4,_ and MnFe_2_O_4_ we employed essentially the same procedure. For example, 7.6 ml of BE, 1.5 ml of OA, and 3 ml of sHD were added into the three-neck flask and degassed at room temperature for 1 h. After that, the temperature of the mixture was raised to 290 °C. In parallel, 2 mmol of the precursor containing 1.33 mmol of Fe(acac)_3_ and 0.67 mmol of M(acac)_2_ (M = Zn, Co, Ni, or Mn) were dissolved in 2.4 ml of BE and instantly added to the hot reaction mixture. The synthesis was carried out for 30 minutes at 290 °C. The obtained product was purified by centrifugation using an ethanol solution.

#### (III) Flow hot-injection protocol of MNPs preparation

The flow hot-injection procedure was implemented in the synthesis of MNPs with two distinct differences in the strategy. In the first attempt, we tested whether using Kim's ratios of chemicals it is possible to get a reasonably well-defined product in terms of morphology. The second one, if (II) proposed fabrication technique allows direct implementation of the above protocol with sHD as a co-solvent. To test it 7.6 ml of BE, 1.5 ml of OA (4.3 mmol) were mixed in a three-neck glass and degassed for 1 hour at room temperature with mechanical stirring. In the case of (II) sHD was added before degassing. Afterward, the solvent or solvent mixture together with OA as an additive was heated to 290 °C under gas flow and stirring. The earlier prepared mixture containing 2 mmol of Fe(acac)_3_ in 2.4 ml of BE was added to the hot solvent(s) under gas flow and injection with a constant speed of 15 ml h^−1^ using a syringe pump. The synthesis was carried out for 45 min from the start of injection and the final product was purified by the same steps as described previously. A list of all prepared samples can be found in Table 1.

**Table 1:** List of samples prepared *via* three distinct strategies heat-up, rapid hot-injection as well as flow hot-injection^a^

Synthesis	Product	Injection	Precursor (mmol)	OA (ml)	sHD (mmol)	Temperature (°C)	Particle size (nm)	SD (nm)
HU	Fe_3_O_4_	—	2	1.5	—	270	48.0	5.3
HU	Fe_3_O_4_	—	2	1.5	—	280	81.3	8.1
HU	Fe_3_O_4_	—	2	1.5	—	290	64.1	6.5
HI	Fe_3_O_4_	Rapid	2	1.3	—	290	72.8	4.7
HI	Fe_3_O_4_	Rapid	2	1.5	—	290	78.7	5.5
HI	Fe_3_O_4_	Rapid	2	1.7	—	290	47.7	4.0
HI	Fe_3_O_4_	Rapid	2	1.5	5	290	47.6	7.5
HI	Fe_3_O_4_	Rapid	2	1.5	10	290	46.6	5.3
HI	Fe_3_O_4_	Rapid	2	1.5	12	290	60.7	7.4
HI	Fe_3_O_4_	Rapid	1.5	1.5	10	290	33.6	5.6
HI	Fe_3_O_4_	Rapid	1.75	1.5	10	290	33.5	3.0
HI	Fe_3_O_4_	Rapid	2.25	1.5	10	290	58	12
HI	Fe_3_O_4_	Rapid	2.5	1.5	10	290	63.6	6.4
HI	Fe_3_O_4_	Rapid	1.75	1.6	10	290	33.3	3.4
HI	Fe_3_O_4_	Rapid	1.75	1.7	10	290	29.9	3.6
FHI	Fe_3_O_4_	Flow	2	1.5	—	290	44.9	5.3
FHI	Fe_3_O_4_	Flow	2	1.5	10	290	49.8	3.3
HI	ZnFe_2_O_4_	Rapid	2	1.5	10	290	40.3	5.0
HI	CoFe_2_O_4_	Rapid	2	1.5	10	290	56.1	7.4
HI	NiFe_2_O_4_	Rapid	2	1.5	10	290	57.2	4.6
HI	MnFe_2_O_4_	Rapid	2	1.5	10	290	51.0	4.7

a HU – heat-up/HI – hot-injection/FHI – flow hot-injection, OA 1.3 ml – 3.7 mmol; 1.5 ml – 4.3 mmol; 1.6 ml – 4.6 mmol; 1.7 ml – 4.9 mmol.

### 2.2 Characterization of materials

Particle size, morphology, and structural properties were evaluated employing transmission electron microscopy using a Tecnai Osiris X-FEG HRTEM microscope operating at 200 kV. MNPs for TEM imaging were prepared by droplet deposition of ethanol-based nanoparticle suspensions (250 μg ml^−1^) on a 200 mesh, carbon-coated copper grid (EM Resolutions United Kingdom). Samples were left for 24 h for complete evaporation of solvent at room temperature and under dust protection. Analysis of the results was performed using ImageJ freeware software (v. 1.8.0_172). Quality of cubic morphology was estimated using so-called cubicity parameter describe as follows:1
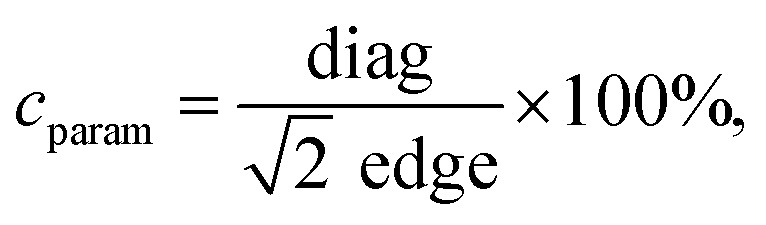
where *c*_param_ is cubicity (value as close to 100% matches with perfect cube shape), diag is simply the diagonal and edge means the edge length of particle.

X-ray powder diffraction technique (XRD) was used to determine the crystal structure of final products with a Bruker D8 Advance diffractometer with the Cu lamp (*K*_α1_: 1.54060 Å) and Ni filter for removal of *K*_α2_ induced reflections. Obtained diffraction patterns were compared with the ferrite standards from the crystal structure database ICDD. Fourier transform infrared spectroscopy technique with a Thermo Scientific Nicolet iZ10 FT-IR-ATR spectrometer was used to study the state of particle surface within the spectral range covering 4000–500 cm^−1^ at room temperature.

## 3. Results and discussion

### 3.1 Effect of the reaction temperature on MNPs

**Fig. 1:**
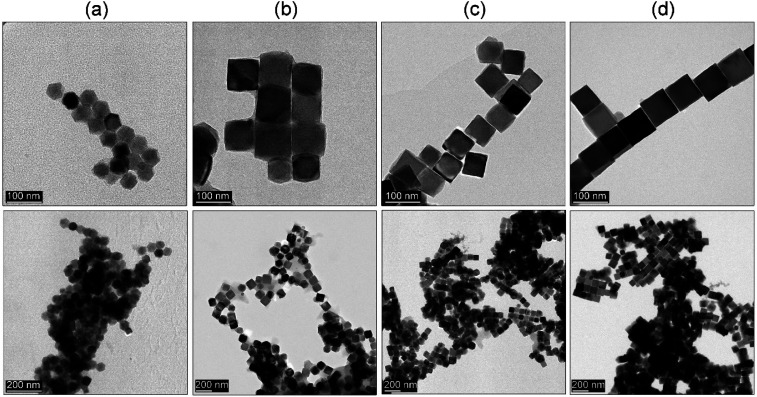
TEM characterization of the heat-up synthesis (a–c) as a function of reaction temperature (a) 270 °C, (b) 280 °C, (c) 290 °C, and (d) rapid hot-injection at 290 °C. The duration of the synthesis was 30 min in each case.

Reference Fe_3_O_4_ MNPs were fabricated through repetition of the simple and efficient one-pot synthesis based on the heat-up approach proposed by Kim *et al.*^23^ at 290 °C with the heating rate of 20 °C min^−1^. As it was expected fixing such reaction conditions and the ratio of all chemicals leads to the formation of the 64 nm nanoparticles with predominant cubic morphology. They proved that it is possible to control the particle size down to 20 nm by a change of the concentration of the iron precursor in the reaction mixture. However, the effect of the reaction temperature was rarely studied.^45^ Thus we decided to perform experiments to check if this important parameter has any effect on particle shape as well. Because of possible BE instability at high temperatures, this seemed to be a very important idea. The dependence of the reaction temperature (270–290 °C, 30 min) has been shown in Fig. 1a–c.

As one can observe the MNPs obtained at 270 °C tend to form tetradecahedrons with an average particle size of 48 nm. Temperature increase to 280 °C leads to the shape transformation into truncated cubes (81 nm, cubicity 93%) whereas synthesis performed at 290 °C resulted in the final product constituted of very well-defined cubes (64 nm) (cubicity 96%). Surprisingly, in comparison with the work of Ding *et al.*,^45^ we observed completely contradictory results upon the use of basically the same experimental procedure (reverse sequence of shape tailoring). Moreover, we tested even lower reaction temperature 260 °C while keeping the same reaction time and a fixed ratio of all substances. No defined shapes were present throughout the sample, not even to mention that the amount of the MNPs fabricated under such conditions was immensely low. The explanation for that can be that the OA concentration-effect increases the decomposition and nucleation temperature.^22,38^ Nonetheless, we have shown that it is possible to use a lower temperature to achieve some additional Fe_3_O_4_ morphologies. Following a very important works of Qiao *et al.*^34^ and Muro-Cruces *et al.*^35^ the particle growth can be directed through the control of the chemical potential of monomers (*μ*_m_) defined as:

2 *μ*_m_ = *μ*^0^_m_ + *RT* ln[*C*_m_*γ*_m_],

where *μ*^0^_m_ describes the standard chemical potential of the monomers at reference state (constant), *R* stands for the ideal gas constant, *T* is the absolute temperature at which reaction is conducted, *C*_m_ is monomer concertation and finally, *γ*_m_ is the activity coefficient of the monomer in the given solution. This thermodynamic equation takes into account the most important parameters affecting the chemical potential of the monomers and can help with the understanding of how the nucleation process and particle growth can be influenced. This is a very strong tool in the engineering of the particle shape. Qiao *et al.*^34^ proposed dependence where the balance between chemical potential of the monomers and chemical potential of crystallographic facets determines the direction of growth with following ranking *μ*_{100}_ > *μ*_{110}_ > *μ*_{111}_. To control particle shape change of the chemical potential of monomers is mandatory to favour a specific growth direction. Therefore, when *μ*_m_ is higher than the chemical potential of *μ*_{111}_ but lower than *μ*_{100}_, *μ*_{110}_ the growth of the {111} facets and formation of cubes will be preferred.^34^ Of course, the role of the additives like OA or solvents cannot be omitted since they effectively affect the behaviour of the monomer in terms of its thermal stability, concentration as well as activity. It is then reasonable that the MNPs prepared as a function of the temperature grow into the tetradecahedrons at 270 °C as a result of the continuous growth along {110} and {111} while growth of the {100} facets under such conditions is inhibited (the *μ*_m_ is lower than *μ*_{100}_). An increase of the temperature up to 280 °C favours growth along {111} direction leading to the change of shape into truncated cubes. Further increase of reaction mixture temperature to 290 °C results in the formation of very well-defined cubes due to the growth of the same facets. We believe that the reaction temperature (fixed parameters and OA as the critical ligand) will mostly enhance the diffusion coefficient of monomers on heavily packed and dense {111} facets facilitating ligand expel and final deposition of the monomer. This cannot be done effectively at lower reaction temperature (higher energy barrier to overcome),^34^ thus different morphologies are formed under a given reaction temperature.

### 3.2 Hot-injection technique as an alternative to heat-up protocol

**Fig. 2:**
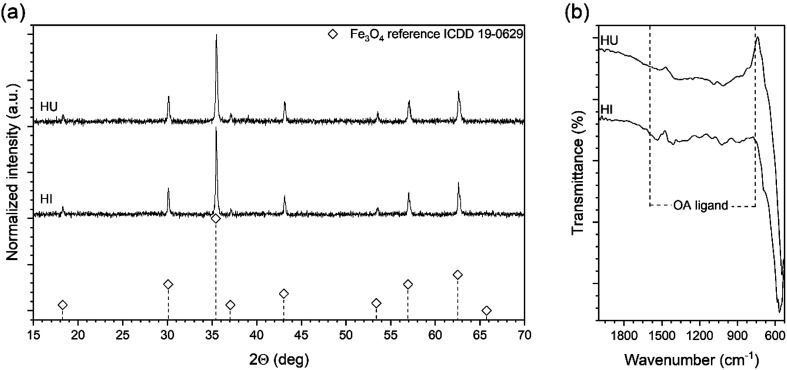
X-ray powder diffraction pattern and FTIR spectrum of the Fe_3_O_4_ MNPs fabricated through heat-up (HU) and hot-injection (HI) techniques.

Based on the results from the previous section we decided to check whether it would be possible to get similar results. We adapted a hot-injection approach which we consider as an alternative limiting the necessity of the BE expose for prolonged action of high temperature. The iron precursor was rapidly injected directly into the hot BE (290 °C) containing the same amount of OA as previously. The reaction mixture was left for the next 30 minutes at 290 °C under a blanket of N_2_ and constant mechanical stirring. As one can see (Fig. 1d) instant injection of the precursor leads to the formation of very well-defined cubic Fe_3_O_4_ nanoparticles (cubicity 98%) assembled into long chains due to the magnetic interactions. Upon comparison with the heat-up technique, hot-injection fabricated particles seem to have more regular cubic morphology but were slightly larger (78 nm). Monodispersity of the MNPs has been also retained in the rapid hot-injection (±6.5 nm for the heat-up and ±5.5 nm in hot-injection). This is a very interesting result since literature lacks any other reports on shape control with exception of Ho *et al.*^29^ work. They reported that slow, flow-injection of the precursor led to the slightly truncated cubes (around 20 nm). However, in their strategy, different quantities of OA were used with the addition of OAm as a particle size reducing and non-specific ligand (no preference to crystallographic facets attachment). Besides tetradecanediol (TDD) as a co-solvent enhancing polarity of the reaction mixture was taken to increase the activity of monomers. As we mentioned earlier the mechanism of the nucleation and particle growth will be quite different and reminds rather a continuous nucleation and seed-mediated growth. Fabrication of fully developed cubic Fe_3_O_4_ involved prolonged time up to 2 h which could be a serious drawback in terms of well-known BE instability at high temperatures affecting reproducibility. We have shown that through the rapid hot-injection process it is enough to leave the reaction mixture at 290 °C for 30 min after precursor injection to obtain cubes while keeping restricted ratios of all substances and process parameters. The structural properties of MNPs prepared using heat-up and hot-injection techniques were checked using the XRD (Fig. 2). The obtained diffraction patterns showed typical for the ferrite family reflections perfectly matched with the reference standard card no. ICDD 19-0629 ascribed to the cubic crystal structure belonging to the *Fd*3̄*m* (no. 227) space group. We did not observe any other reflections confirming that both studied samples are single phase. The FTIR spectrum revealed presence of the residual organics on the surface of the nanoparticles synthesized *via* all protocols. Peaks located at characteristic positions namely 937, 1285, 1409, 1462 cm^−1^ were attributed to the oleic ligand. Typical band at 591 cm^−1^ corresponds to the strong vibrations of the Fe–O bonds.

### 3.3 Effect of the OA ligand on the directional growth of MNPs

**Fig. 3:**
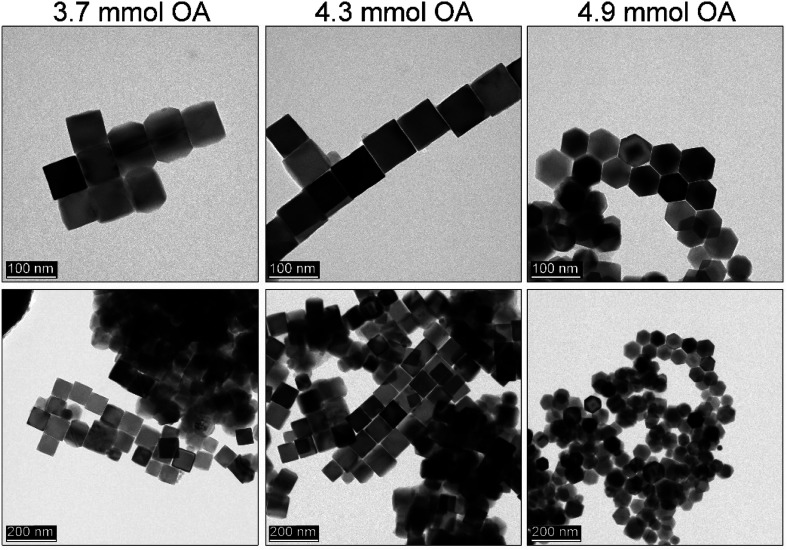
Effect of the different OA content on the morphology of the Fe_3_O_4_ MNPs fabricated *via* rapid hot injection technique performed at 290 °C for 30 min (from left to right 1.3 ml, 1.5 ml and 1.7 ml of OA).

Since the idea of the rapid hot-injection worked very well in a synthesis of morphologically controlled MNPs we have been particularly interested if the OA content (1.3–1.7 ml; 3.7–4.9 mmol) is within optimal range for the particle shape tuning (see Fig. 3). The amount of the iron precursor was fixed at 2 mmol in the injection syringe (total volume 2.4 ml). Interestingly, we have found that when the volume of the OA is below 1.5 ml truncated cubes are predominantly present (cubicity 92%) with a fraction of other morphologies. The use of the OA up to 1.5 ml seems to be optimal for the growth of cubic particles. Whereas the addition of 1.7 ml of OA results in the formation of well-defined tetradecahedrons. We are convinced that such behaviour is caused by an increase in the polar character of the reaction mixture. This is due to a higher activity of the monomer and consequently, a change of the chemical potential of monomers that leads to the preferred growth along {100} and {110} directions.^34^ We also found that the OA amount within used range of 1.3 to 1.5 ml did not cause a significant difference in particle size (around 70–80 nm). While at 1.7 ml of the OA MNPs size decreased down to 48 nm. This can be an indication of a change of monomer diffusion and inhibition of particle growth. Therefore, we concluded that if cubic particles are of strategic aim the optimal content of the OA in the reaction mixture should be fixed at 1.5 ml (4.3 mmol) at given concentration of Fe(acac)_3_.

### 3.4 Effect of the saturated hydrocarbon co-solvent on the MNPs size and morphology

**Fig. 4:**
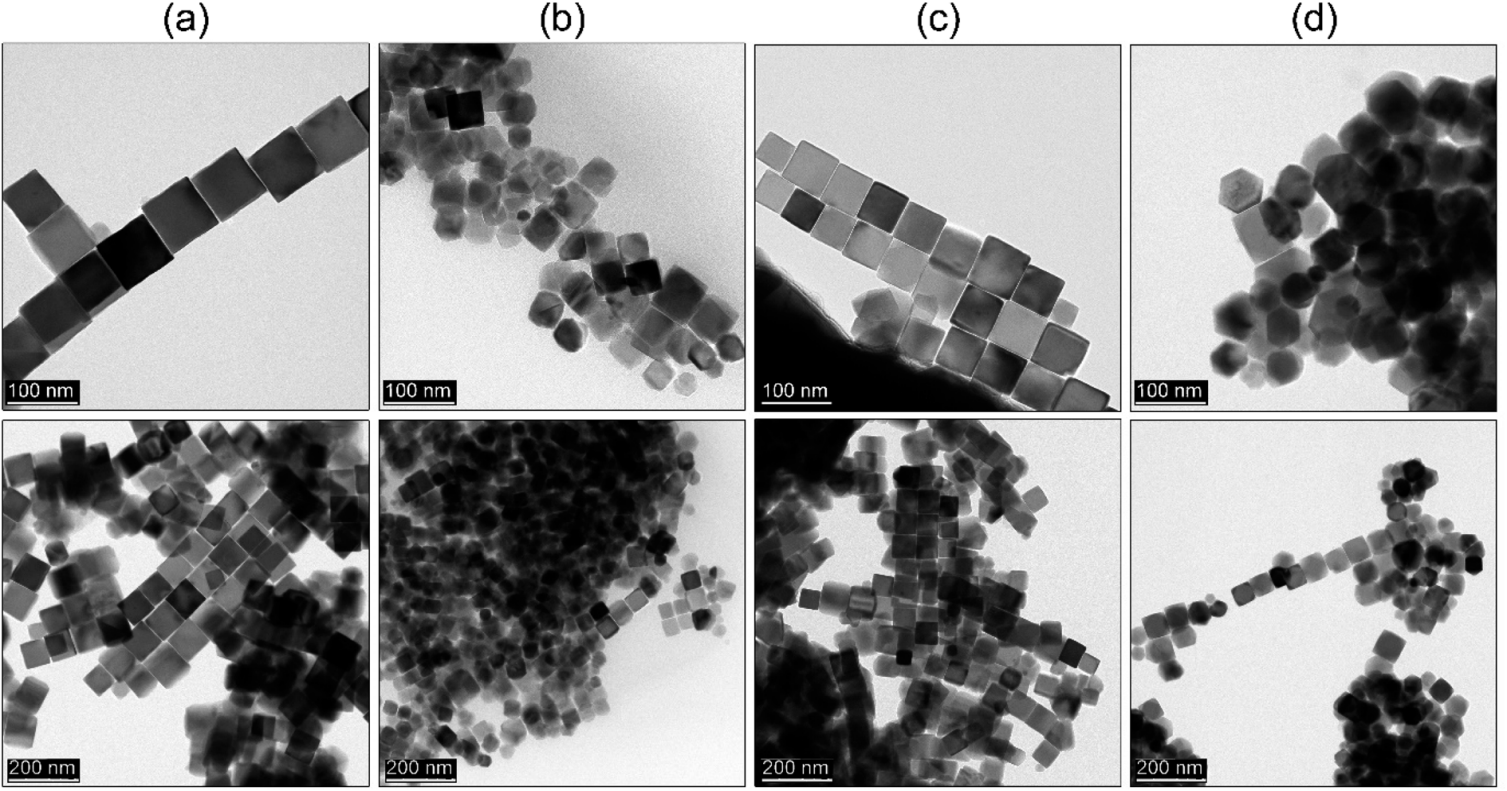
Effect of the addition of the strongly non-polar co-solvent on Fe_3_O_4_ nanoparticles morphology synthesised by the rapid hot-injection technique, (a) without sHD, (b) 5 mmol of sHD, (c) 10 mmol of sHD, and (d) 12 mmol of sHD.

Another challenge was to carry out trials on particle size control while maintaining product morphology. This is a key issue since for several applications the dimension of particles might be too large. We decided that the best solution would be a drastic change of the activity coefficient of monomers while keeping other synthetic parameters constant *i.e.* reaction temperature set to 290 °C, time 30 min, and 1.5 ml of OA. The best way to limit the monomer activity is to choose a co-solvent with a distinctly different chemical character inducing a change in interactions between monomer and solvent molecules. Since the iron acetylacetonate has a polar character this can be done by the addition of strongly non-polar solvents as reported for the heat-up approach.^34,35^ Therefore, hexadecane (saturated hydrocarbon with a high boiling point) was proposed by us as a co-solvent to inhibit the monomer activity coefficient. The results of the MNPs fabrication as a function of the sHD content is shown in Fig. 4. Indeed, the addition of the sHD affects the particle size. Whereas a high amount of sHD suppresses the activity coefficient of monomers that much, that it is not possible to tailor a particle shape. Based on the TEM analysis we found that downsizing of the MNPs with sHD can be achieved to 46 nm while keeping cubic morphology (cubicity 99%) for a maximum of 10 mmol of sHD in the reaction mixture (Fig. 4c). Further increase of the sHD led to the uncontrolled growth of particles.

### 3.5 Effect of the monomer concentration on MNPs and further size tuning with OA

**Fig. 5:**
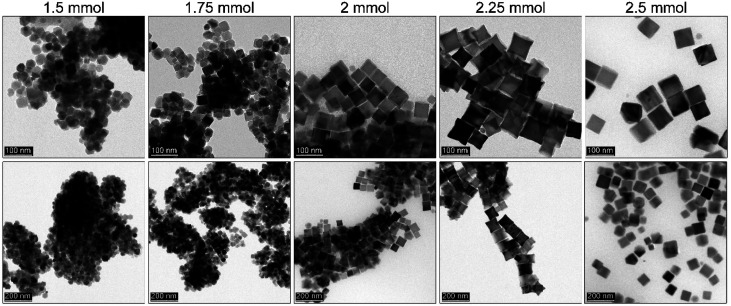
Dependence of the iron precursor content on the Fe_3_O_4_ MNPs obtained by thermal decomposition *via* a rapid hot-injection approach.

It is very well known that the precursor concentration has a tremendous effect on the nucleation and particle growth stage. A balance has to be found to control the particle size and shape. Therefore, it was of great importance to optimize this parameter since the nucleation process is instantaneous and the mechanism of the particle formation is different from that of a heat-up approach. Since the iron precursor concentration was quite high, we wanted to demonstrate whether after optimization of the reaction temperature, OA, and sHD it is possible to limit precursor content. This could assure some additional flexibility in further size tuning without a change in morphology (Fig. 5). The amount of the iron acetylacetonate checked varied from 1.5 mmol to 2.5 mmol keeping constant volumes in both injection syringe and reaction mixture.

We observed that the lowest concentration of the iron precursor leads to the formation of the smallest particles (30 nm) with a polyhedral morphology. Low precursor concentration causes difficulties with shape control due to an insufficient number of monomers needed to terminate directional growth. A slight increase of precursor up to 1.75 mmol forces the formation of the truncated cubes with an average particle size of 33 nm, whereas 2 mmol of Fe(acac)_3_ seems to be enough to tune cubes. We found that in the latter case particles tend to grow to bigger sizes (46 nm). Further increase of the iron source results in {111} vertices growth leading to the star-like particle morphology (46 nm). According to the literature data growth towards star-like structures can be obtained for relatively high monomer concentration. The particle size distribution up to the 2.25 mmol of the precursor remains narrow leading to the monodisperse particles. However, at 2.5 mmol 63 nm cubes (cubicity 99%) can be individuated with the presence of much smaller objects. It suggests that the extent of monomers which cannot be consumed during growth leads to formation of new particles.

**Fig. 6:**
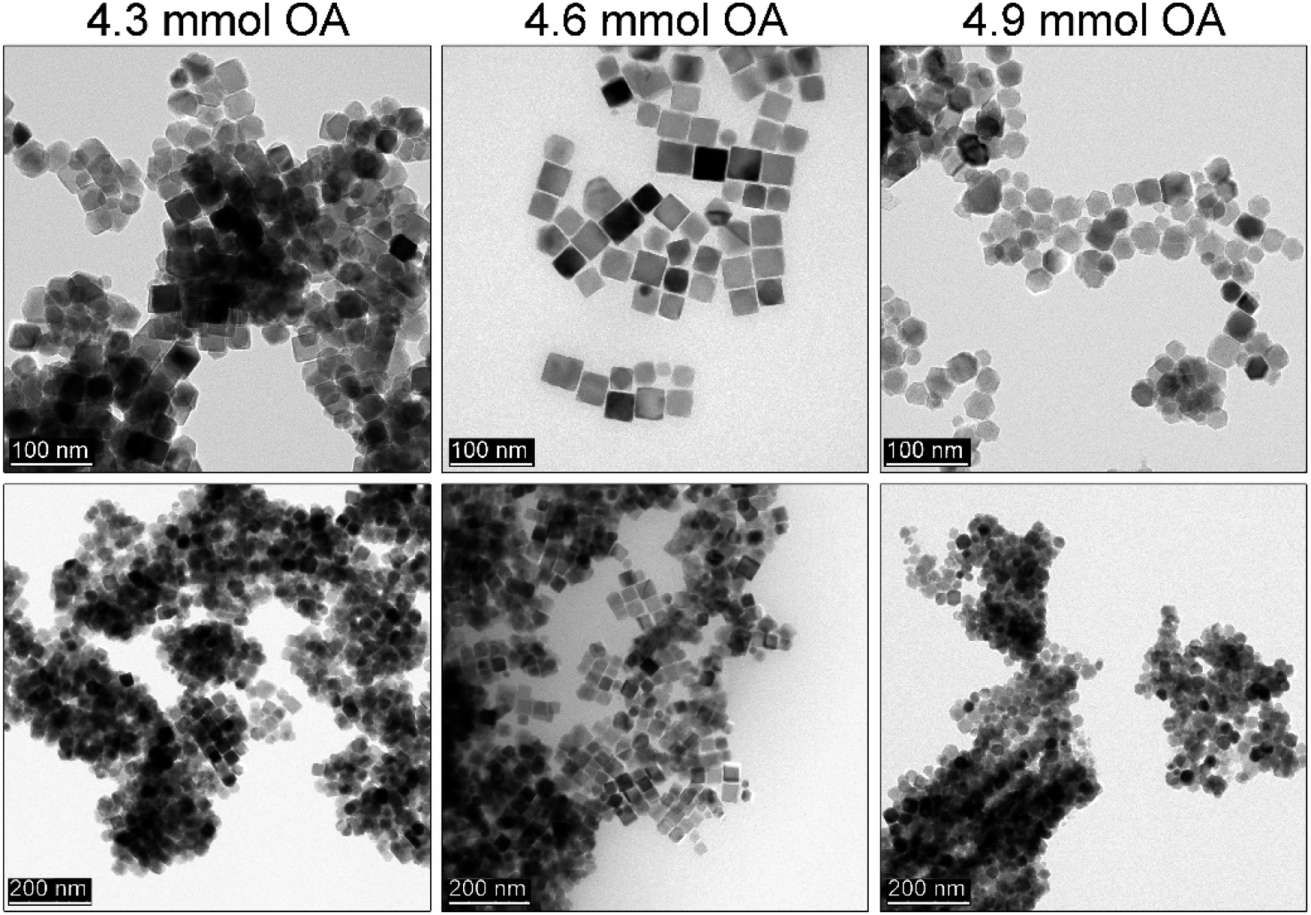
Additional OA effect on the morphology of the Fe_3_O_4_ MNPs synthesized by the rapid hot-injection with optimal content of the sHD (10 mmol) co-solvent and 1.75 mmol of the iron precursor (from left to right 1.5 ml, 1.6 ml and 1.7 ml of OA).

All of these observations are in agreement with expected behavior. The lower concentration of monomers will result in unfinished directional growth of particles that form polyhedras. Particles remain relatively small and cannot grow further due to the quenched reaction (short synthesis time). Higher precursor content leads to better control over morphology and at maximum amount used here causes secondary particle growth. Following Kwon and Hyeon,^44^ the mechanism of particle formation in the hot-injection relies on a fast nucleation reaction which stops at a supersaturation level. Particle growth is diffusion-controlled that allows for so-called size focusing resulting in a monodisperse product. To tune further the particle size 1.75 mmol of the iron precursor was used with slight OA (1.5–1.7 ml, 4.3–4.9 mmol) adjustments. We found that (see Fig. 6) in comparison to the 1.5 ml OA the addition of extra 0.1 ml (1.6 ml) resulted in mastered cubes (cubicity 93%). However, such a small rise in ligand content did not cause a size change but facilitated directional growth. Further increase of the OA led to the size reduction (29 nm) and progressing issue with particle morphology (polyhedral shapes) as a consequence of the change of monomers chemical potential. An important conclusion can be drawn that control of the particle size and shape depends on many parameters as in the case of the heat-up. However, utilization of the rapid hot-injection protocol proposed by us allows for the control of the MNPs particle size (to some extent) and morphology through modification of synthetic parameters.

### 3.6 Effect of the flow-injection process on MNPs

**Fig. 7:**
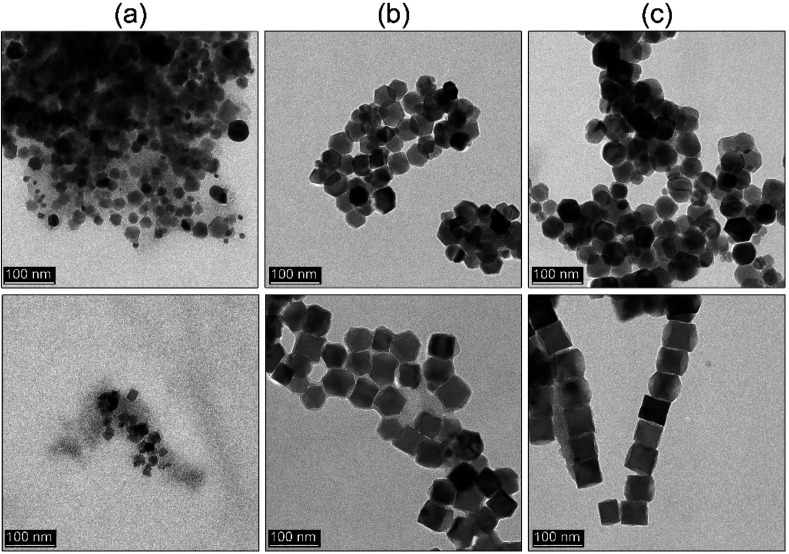
Flow-injection technique as an alternative to rapid injection. Upper panel MNPs fabricated without sHD, a bottom panel with sHD, the injection speed was 15 ml h^−1^. Time evolution of particle shapes measured after finished flow-injection (a) 15 min, (b) 30 min, (c) 45 min.

Ho *et al.*^29^ showed that it is possible to prepare shape-controlled Fe_3_O_4_ through constant flow hot-injection. Therefore, we tried to implement our strategy directly and see if Ho modification can be used for tuning MNPs morphology using our protocol. The main difference is that both strategies are based on different chemicals, their ratio, and Ho did not use a co-solvent as well. The reaction temperature kept the same (290 °C), the flow rate was set at 15 ml h^−1^ and the process was carried out for 45 min measured from the start of the flow-injection of monomer. We did a comparison with the procedure without use of the sHD (details can be found in the Experimental section) to see if it still plays an anticipated role (see Fig. 7). Interestingly, the flow-injection without and with sHD shared common features. The first sign of product formation was observed after 15 min, whereas particle morphology tends to be defined within 30 min. In both approaches, the particle size was around 45–49 nm. We found noticeable differences as well. Both product morphologies are distinctly different due to the action of the sHD. In the case of the flow-injection where sHD was not added MNPs tend to grow into polyhedral shapes after 30 min. The growth of particles is already finished since there is no significant particle size increase after 45 min. The presence of non-polar co-solvent (sHD) led to the formation of mixed morphology after 30 min. After additional 15 min particles transformed into cubes with a size of 49 nm. Definitely, the sHD strongly limits the activity of monomers forcing directional particle growth. A comparison with the rapid hot-injection is striking since the growth of the cubic MNPs is already finished after 30 min. The same process of particle shape molding in the flow-injection takes a longer time. This points out a significant difference in the mechanism of particle formation. The directional growth of the particles is a function of the additive/co-solvent and iron precursor ratio, speed of the flow-injection, synthesis time, and process temperature affecting chemical potential. All discussed approaches *i.e.* heat-up, rapid hot-injection, as well as flow-injection, can be used as tools for the control of the particle size and morphology leading to highly monodisperse MNPs. We have shown that the rapid hot-injection technique due to its simplicity can be successfully applied in the synthesis of the well shape-defined MNPs. The rapid hot-injection technique leaves space for particle size and shape control. Moreover, synthesis duration can be significantly reduced to 30 minutes, thus the risk of thermal instability of the BE can be limited to the absolute minimum.

### 3.7 Hot-injection approach towards other MFe_2_O_4_ ferrites

**Fig. 8:**
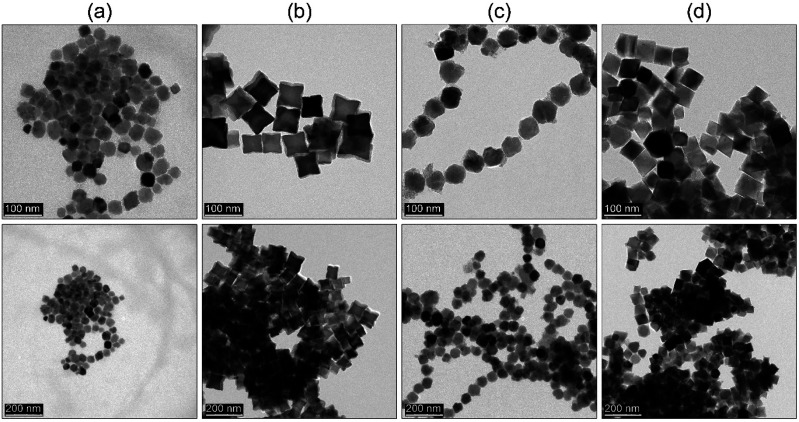
TEM images of (a) ZnFe_2_O_4_, (b) CoFe_2_O_4_, (c) NiFe_2_O_4_, and (d) MnFe_2_O_4_ nanoparticles synthesized by thermal decomposition *via* hot-injection method.

Lastly, we checked whether the proposed approach can be further extended to other ferrite representatives (ZnFe_2_O_4_, CoFe_2_O_4_, NiFe_2_O_4_ and MnFe_2_O_4_) using the same rapid hot-injection procedure without any other modifications (see Fig. 8). In the case of the ZnFe_2_O_4_ and NiFe_2_O_4_ nanoparticles, we found that optimization is mandatory since ZnFe_2_O_4_ and NiFe_2_O_4_ particle shape tends to be a mixture of polyhedral morphologies with the size around 40 and 57 nm. In the case of the CoFe_2_O_4_ and MnFe_2_O_4_ compounds, both products formed star-like and octahedral particles, respectively with comparable size (around 56 nm). Such behaviour might be caused by different temperature stability of the metal complexes. Cobalt and iron acetylacetonates have comparable stability. Therefore, their intermediate complexes should behave similarly. This led to the directional growth of the {111} facets towards cubes and stars. The manganese precursor has the highest temperature of decomposition (250 °C). Thus, most likely the rate of nuclei formation will be slower and octahedral particles are predominantly present due to that growth occurs faster along {100} and {110} directions so particle morphology will resemble the shape of primary nuclei.^1^ The NiFe_2_O_4_ ferrite, since nickel precursor has its decomposition temperature between cobalt and manganese complexes so for that reason intermediate morphology was found. Whereas in the case of ZnFe_2_O_4_ we found that it contains mixed morphologies. One of the main reasons for that could be seen in the presence of water in the precursor (a hydrated form of zinc acetylacetonate was used due to the unavailability of a non-hydrated form in the market). So the result has been attributed to the detrimental effect of water molecules on particle shape that led to the formation of irregular particles.^35^

## 4. Conclusions

We have shown that the rapid hot-injection technique can be alternatively used for morphology (octahedrons, cubes, and stars) and particle size controlled MNPs at a relatively short synthesis time (30 min) minimizing the risk of main BE solvent decomposition. In contrast to the heat-up technique, the rapid hot-injection method is a single step and does not need careful control over the heating rate of the reaction mixture. The process of shaping particle morphology is complex and strongly depends on the synthesis parameters (temperature, concentration of precursor, additives, co-solvent, *etc.*). The use of strongly saturated hydrocarbons as co-solvent *i.e.* hexadecane (sHD) instead of long-chain diols (tetradecanediol – TDD; or hexadecanediol HDD) in the presence of OA additive changes the molecular interactions between solvent and monomer and allows for particle shape control. We postulated that the addition of the sHD co-solvent to the complex reaction mixture changes the monomer activity coefficient, reduces the chemical potential of monomers, and thus directional growth along specific facets can be achieved. We have shown, that it is also possible to produce shape-controlled particles *via* the flow-injection process by using the protocol proposed by us but with the necessity of further optimization to master morphology and particle size. The main advantage of the HI approach with sHD co-solvent as monomer activity limiting agent lays in the possibility of synthesis time reduction (down to 30 min), minimizing the risk of dibenzyl ether degradation upon prolonged exposure to high temperature, use of less complex apparatus without the necessity of temperature ramp control. Elimination of long-chain diols in favor of sHD can protect from possible water release upon side reactions leading to uncontrolled growth of particles during the HU approach. We emphasized that there is a high demand for finding a better solvent than BE since its instability at high temperatures is a serious source of issues with product reproducibility upon longer synthesis time.^35^

## Conflicts of interest

There are no conflicts to declare.

## Supplementary Material
